# Polysomnographic arousal index and periodic limb movement index in patients undergoing coronary artery bypass grafting: an exploratory case-comparison study

**DOI:** 10.1007/s11325-026-03779-9

**Published:** 2026-08-06

**Authors:** Jaidaa Mekky, Henyah W. Dardir, Kaitlyn Malek, Ahmed Adham R Elsayed, Sherif W. Ayad, Ismail Ramadan, Horeya Saadallah, Marc D. Basson

**Affiliations:** 1 Neuropsychiatry Department, Faculty of Medicine, Alexandria University, Alexandria, Egypt; 2 Cardiology Department, Faculty of Medicine, Alexandria University, Alexandria, Egypt; 3 College of Medicine, Northeast Ohio Medical University, Rootstown, OH 44272 USA; 4 Department of Surgery, Northeast Ohio Medical University, Rootstown, OH 44272 USA; 5 Department of Biomedical Sciences, Northeast Ohio Medical University, Rootstown, OH 44272 USA

**Keywords:** Obstructive sleep apnea, CABG, CAD, Sleep disorders

## Abstract

**Purpose:**

Obstructive sleep apnea (OSA) is a known risk factor for coronary artery disease (CAD), but its phenotypic variations and their impact on cardiovascular risk remain unclear. Recent studies suggest that a high periodic limb movement index (PLMI) and a high arousal index OSA phenotype may contribute to cardiovascular disease. This study aims to assess the association between this phenotype and CAD in patients undergoing preprocedural screening for coronary artery bypass grafting (CABG).

**Methods:**

This case-comparison study analyzed a prospective adult patient group undergoing polysomnography before CABG, compared to an age- and gender-matched retrospective group undergoing polysomnography for employment screening. Sleep parameters, including PLMI, arousal index, apnea-hypopnea index (AHI), and sleep onset latency, were assessed. Statistical analysis, including chi-square tests, Fisher’s exact test, and multivariable logistic regression, was conducted to evaluate associations between sleep disturbances and CAD.

**Results:**

CAD patients had significant differences in the amount of abnormal PLMI, arousal index, and AHI values compared to the non-CAD group (*p* = 0.0002, *p* = 0.002, and *p* = 0.015, respectively). Multivariable regression identified high PLMI and arousal index as the strongest associations of CAD.

**Conclusion:**

A high PLMI/high arousal index OSA phenotype may increase cardiovascular risk. Identifying high-risk OSA phenotypes could improve early CAD screening and intervention strategies. Further research is needed to explore underlying mechanisms.

**Graphical abstract:**

Created in BioRender. Dardir, H. (2025) https://BioRender.com/9ifvxgl.

**Figure:**
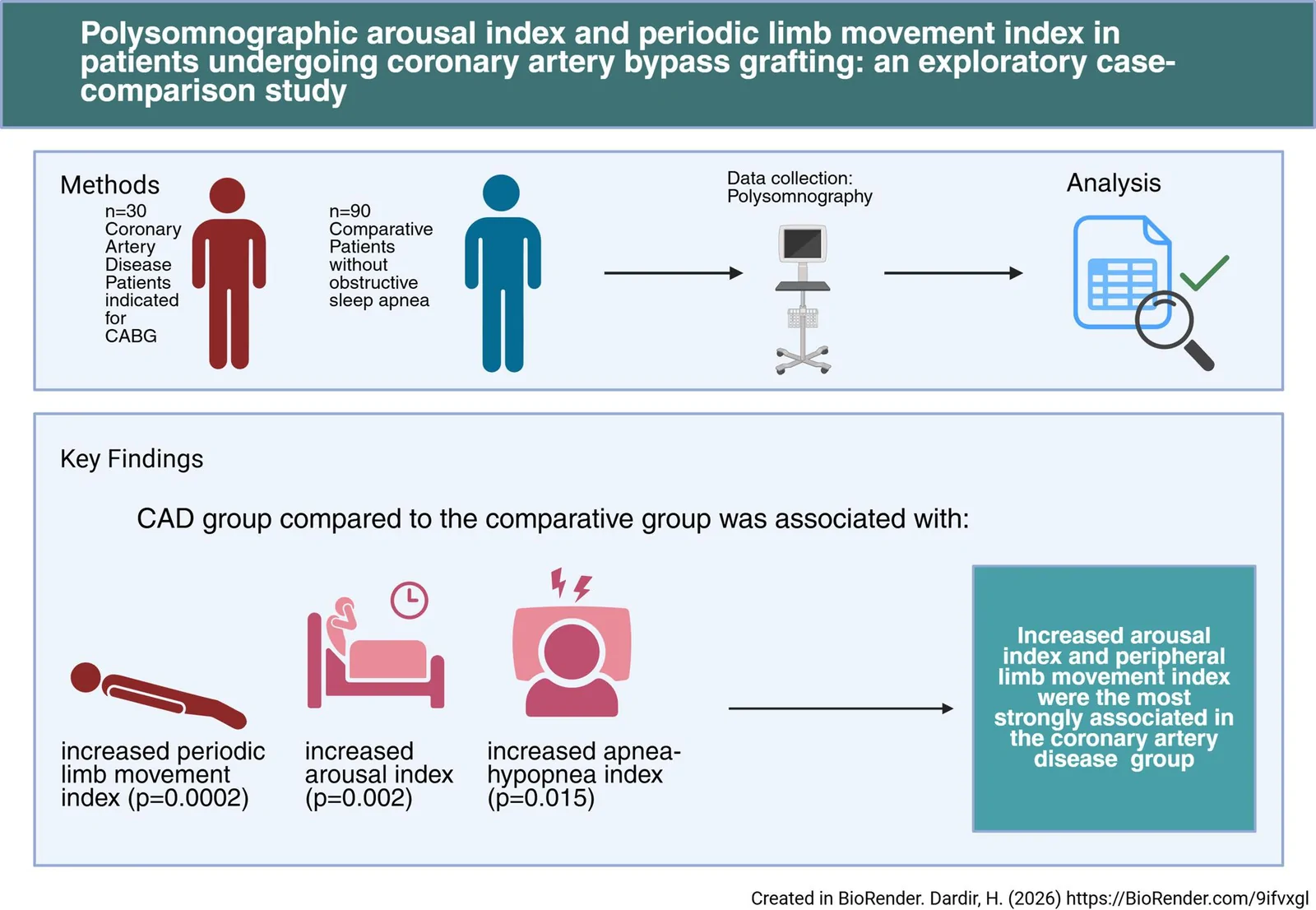

**Supplementary Information:**

The online version contains supplementary material available at 10.1007/s11325-026-03779-9.

## Introduction

Research has long established a connection between obstructive sleep apnea (OSA) and coronary artery disease (CAD) in the literature [1–4]. OSA is characterized by repeated episodes of partial or complete obstruction of the respiratory passages during sleep [5]. OSA is, however, increasingly being recognized in recent years as a complex and heterogeneous disorder wherein different OSA phenotypes may exist [6]. Approximately one billion people globally are estimated to have OSA [7] However, the systematic effects of OSA on physiological systems are still not fully understood. A recent meta-analysis conducted by Yu et al. [8] has shown that the gold standard OSA treatment of nocturnal CPAP utilization does not significantly decrease cardiovascular disease risk associated with OSA. This necessitates an increased understanding of the disease pathology that links OSA with cardiovascular disease.

Recent research suggests that periodic limb movement in sleep (PLMS), a phenomenon commonly observed in OSA patients, may contribute to cardiovascular risks associated with the disease. Periodic limb movement in sleep is characterized by involuntary, repetitive limb movements during sleep, often leading to sleep fragmentation and arousal [9]. The prevalence of periodic limb movement in sleep is estimated to be 4–11% in adults [10, 11].While traditionally treated as an incidental finding in OSA, newer studies have suggested that increased periodic limb movement in sleep frequency may be directly correlated to sympathetic nervous system activity and potentiate a greater risk of cardiovascular hypertension and arrhythmias [12]. In fact, the presence of periodic limb movement in sleep in OSA patients has been associated with lower CPAP adherence, potentially due to unresolved sleep disruptions that persist despite airway-stabilizing interventions [13].

There is growing interest in defining OSA phenotypes that are at increased risk of cardiovascular disease. Currently, there is speculation that a higher periodic limb movement index/high arousal index phenotype of OSA may be concurrent with an increased likelihood of developing CAD [14]. Arousal index, defined as the frequency of awakenings per hour, is a particularly notable covariate as it has been suggested to reflect increased autonomic instability, promoting long-term cardiovascular risk [12].

This study aims to further elucidate the relationship between OSA, PLMS, and CAD by analyzing polysomnographic parameters in patients undergoing preprocedural screening for coronary artery bypass grafting (CABG). By determining whether a high periodic limb movement index/high arousal index phenotype is disproportionately represented in the CAD patient group, we seek to demonstrate further linkage between sleep disturbances and cardiovascular disease.

## Methods

### Design and participants

Patients were categorized into two groups: a prospective case group of 30 consenting patients with angiography-confirmed coronary artery disease (denoted as “CAD”) who were referred for CABG. These patients consented to undergo preoperative polysomnography prior to their scheduled CABG and are referred to as the CAD group. A retrospective comparative group (denoted as “C”) of 197 patients without coronary artery disease who were referred to polysomnography testing for reasons other than OSA, including suspected parasomnia, occupational requirements, or shift work sleep disorder. These comparative group patients undergoing screening had not been previously screened for OSA and did not have a previous OSA diagnosis. Three-to-one propensity matching was done between the comparative group and the CAD group, which then yielded a comparative group of 90 patients from the 197 comparative patients to be utilized in the study. All CAD group patients were indicated for CABG and had CAD in three or more vessels with 70% or greater occlusion. The study was approved by the Institutional Review Board of both Alexandria Faculty of Medicine and Northeast Ohio Medical University.

### Polysomnography

The PSG study was conducted using the Cadwell data acquisition system (Version 2.1, USA) at the sleep laboratory of the Department of Neuropsychiatry at Alexandria University Hospital. The PSG recordings included four EEG channels, two lateral canthus eye leads for electrooculogram (EOG), two submental electromyography (EMG) leads, two electrocardiogram (ECG) leads, one nasal thermistor for airflow, one channel for oxygen saturation (SO2), chest and abdominal belts for monitoring respiratory movements, and two anterior tibialis EMG leads. Two independent sleep specialists scored the PSG data according to the American Academy of Sleep Medicine (AASM) Standard Manual for scoring sleep-associated events (2020). The sleep parameters collected for analysis included sleep efficiency, sleep latency, arousal index, and total apnea-hypopnea index (AHI). Additionally, the study recorded the periodic limb movement index.

### Statistical analysis

**Fig. 1:**
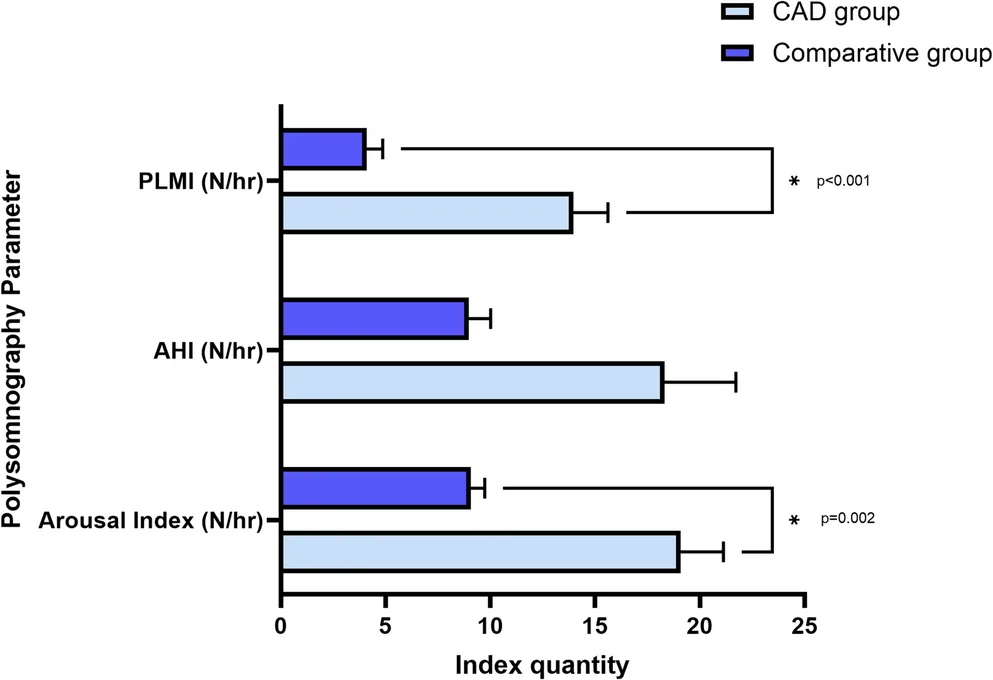
Mann-Whitney test Of AHI, PLMI, arousal index in the coronary artery disease (CAD) group indicated the CABG procedure vs. the comparative group

Patients were propensity matched based on age and gender with a 1:3 matching of CAD: Comparative group to reduce confounding. Propensity score matching was conducted in XLSTAT Premium statistical software using the propensity match algorithm to estimate the probability of CAD group assignment based on age and gender. A 1:3 nearest-neighbor matching without replacement was applied using the logit of the propensity scores, with a caliper of 0.10 times the standard deviation to limit unreasonable matches. Values of the CAD group and the comparative group prior to propensity matching are shown in Supplemental Table 1. Testing for a Gaussian distribution was done using the Shapiro-Wilk test. Non-normally distributed data was represented by the Shapiro-Wilk test significance (*p* < 0.05). Chi-square analysis was utilized to determine significant associations between case-comparative grouping and the outcomes of abnormal sleep onset latency, sleep efficiency, arousal index, periodic limb movement index (PLMI), and Apnea-Hypopnea Index (AHI). For Chi-square theoretical values less than 5, Fisher’s exact test was utilized for the same determination of significance. The Mann-Whitney rank sum test was used for data with a non-normal distribution. A multivariable logistic regression analysis was performed including AHI, PLMI, and arousal index. Variance inflation factors (VIFs) were calculated to assess multicollinearity among the predictors (Supplemental Table 2). The probability value of *p* < 0.05 was considered statistically significant. Statistical analyses were conducted using XLSTAT premium statistical software (Lumivero, Denver, CO) and GraphPad Prism 10.4.0 (Dotmatics, Boston, MA). The standard error of the mean was utilized to generate Fig. 1. 

### Polysomnography parameters

Sleep onset latency refers to the duration of time taken for an individual to transition from wakefulness to sleep once the test begins, with a range of 10–20 min being normal. Sleep efficiency is the proportion of time spent sleeping relative to the total time spent in bed; values at or above 90% are what is considered normal. The arousal index quantifies the frequency of awakenings per hour, where a value greater than 10 is abnormal. The Periodic Limb Movement Index (PLMI) measures the frequency of limb movements during sleep per hour, with an index below 15 being normal, and values above 15 indicating abnormal levels of movement. The apnea/hypopnea index (AHI) assesses the number of apneic and hypopnea events per hour, where an index number greater than 5 is considered abnormal, and an AHI greater than 15 is considered severe/moderate OSA. The AHI index is the main metric for diagnosing OSA.

## Results

### Patient characteristics

Patients were divided into two cohorts: a CAD cohort of 30 patients with coronary artery disease, indicated for coronary artery bypass grafting, who were referred to polysomnography testing, and a comparative cohort of 90 patients who were referred to polysomnography testing for reasons other than obstructive sleep apnea. The mean age of the CAD patient group was 56 (+/- 7.17), and the mean age of the comparative group was 52 (+/-7.22).

### Sleep parameters

The Mann-Whitney test showed that arousal index, periodic limb movement index, AHI, and sleep onset were significantly different in the CAD group compared to those in the comparative group (*p* < 0.0001, *p* < 0.0001, *p* = 0.018, *p* < 0.005, respectively, Table 1). The mean periodic limb movement index for the CAD group was higher compared to the comparative group (14.0 (0.5–31.6) vs. 3.6 (0.5–30.6) Table 1) as well as the arousal index (19.1 (2.5–46.7) vs. 9.2 (0.0–30.0), Table 2) and AHI (18.3 (0.0-70.3) vs. 8.5 (0-28.2), Table 1). Patients in the CAD group exhibited significantly higher AHI (*p* = 0.015, Table 2) and arousal index (*p* = 0.0002, Table 2), as demonstrated using Chi-square testing and higher PLM (*p* = 0.002, Table 2) utilizing Fisher’s exact test compared to the comparative group.

**Table 1:** Subject characteristics and polysomnography parameters in CAD group and comparative group in 1: 3 propensity match

Subject characteristics	CAD group (*n* = 30)	Comparative group (*n* = 90)	*p*
Age	56 (± **7.17**)	52 (± **7.22**)	0.009*
Gender (male)	24 (80%)	50 (57%)	0.001*
PSG Parameters, median (range)			
Sleep Efficiency %	93 (82–99)	90 (47–100)	0.090
Sleep Onset Latency, min	5.93 (1.00-16.50)	11.6 (0–44.0)	0.005 *
AHI, N/hr	18.3 (0.0-70.3)	8.5 (0-28.2)	0.018 *
Arousal Index, N/hr	19.1 (2.5–46.7)	9.2 (0.0–30.0)	< 0.0001*
PLMI, N/hr	14.0 (0.5–31.6)	3.6 (0.5–30.6)	< 0.0001*

*PSG* polysomnography, *PLMI* periodic limb movement index, *CAD* coronary artery disease, *AHI* apnea-hypopnea index

**Table 2:** Chi-square analysis in CAD group and comparative group

PSG parameter	CAD (*n* = 30) *N* (%)	Comparative (*n* = 90) *N* (%)	x² *p*	Fisher’s exact *p*
AHI (N/hr) 16 or higher	16 (53%)	26(28%)	**0.015**	
Sleep Efficiency (%) < 80%	9 (30%)	49 (54%)	**0.020**	
Arousal Index (N/hr) > 10	29 (96%)	62 (69%)	**0.0002**	
PLMI (N/hr) > 15	26 (86%)	21(25%)	*	**0.002**

*Indicates to use Fisher’s exact due to the theoretical value less than 5

*PSG* polysomnography, *PLMI* periodic limb movement index, *CAD* coronary artery disease, *AHI* apnea-hypopnea index

### Multivariable logistic regression

AHI, PLMI, and arousal index were included in a multivariable logistic regression. The CAD group was most correlated with arousal index and total periodic limb movement index (*p* = 0.002, *p* < 0.001, respectively) as shown in Table 3. The results of the multivariable regression showed that high arousal index and PLMI were strongly observed in the CAD group compared to the comparative patient group (β = -0.114, β = -0.125, respectively, Table 3).

**Table 3:** Multivariable logistic regression analysis

	β	*p*	Odds ratio (95% CI)
Arousal index	-0.114	**0.002**	0.892 (0.831–0.958)
AHI	-0.032	0.120	0.968 (0.929–1.008)
Total Periodic Limb Movement Index (PLMI)	-0.125	**< 0.001**	0.883 (0.835–0.934)

*AHI* apnea-hypopnea index

## Discussion

A significant difference in PLMI and arousal index was observed in the CAD group compared to the comparative group across all statistical tests. Multivariable regression also showed that increased arousal index and PLMI were strongly observed in the CAD group, with increased arousal index being the strongest association. Shorter sleep onset latency was also found to be significant; however, this may simply reflect greater sleep pressure from sleep fragmentation rather than an independent predictor.

OSA has been a long-established risk factor in the development of coronary artery disease [2, 15]. Significantly increased AHI in the CABG group reflected this connection between OSA and coronary artery disease. OSA is independently associated with cardiovascular morbidity and mortality and is linked independently to both structural coronary artery disease, depicted by atherosclerosis of the epicardial arteries, and functional coronary artery disease, which is characterized by coronary vasomotor disorders [16]. OSA creates a complex interaction between intermittent hypoxic episodes, sleep disturbance and fragmentation, and intra-thoracic pressure swings to create a maladaptive response that promotes atherogenesis [17]. This atherogenesis, caused by sympathetic overactivity, endothelial dysfunction, chronic inflammation, the generation of reactive oxygen species, and metabolic dysregulation, leads to coronary artery disease [16]. This CAD risk is not necessarily always improved, however, by the gold standard treatment of CPAP for all cardiovascular risk OSA phenotypes, which necessitates further study into the specificities regarding OSA phenotype classification to determine treatment efficacy for individual patients [8, 18].

The heterogeneity of OSA’s manifestation in different patient populations has been increasingly studied concerning OSA phenotypes. Presently, however, there is no consensus for classifying OSA into various phenotypes [19]. Current categorizations of OSA patients have not yet advanced to endotype status to allow for meaningful biomarker and therapy development [6]. Different phenotypes are then distinguished from others by a single or a combination of disease features such as symptoms, response to therapy, health outcomes, and quality of life [6]. Phenotype recognition may have implications for the perioperative health care team, as OSA patients with high arousal thresholds are more sensitive to sedatives and narcotics and carry a higher risk of respiratory distress in the perioperative period [19]. A specific OSA phenotype that has been described is associated with high PLMI related to increased cardiovascular and cerebrovascular disease risk, and that PLMI may be an even greater predictor of cardiovascular risk than an abnormal AHI score [20]. This high arousal index/ high PLMI phenotype may provide a marker for preventative coronary artery disease screening based on past literature and is supported by our observations, where the CAD group had significantly increased arousal index and PLMI in all statistical tests and increased AHI in all tests except multivariable analysis.

Periodic limb movement syndrome is a phenomenon frequently associated with OSA that has been most regarded as an incidental comorbidity to OSA [21, 22]. Recent studies have shown, however, that periodic limb movement syndrome, defined as a peripheral limb movement index of higher than 5 limb movements per hour, may play a significant role in the sleep disruption caused by OSA in patients and is a defining trait of a high PLMI/high arousal index phenotype of OSA that has an increased risk of cardiovascular and cerebrovascular disease [20, 21]. Studies also suggest that the increased periodic limb movement in this high PLMI/high arousal index phenotype could be a result of increased basal sympathetic predominance, which could negatively impact the cardiovascular system of OSA patients [23]. This apparent link between PLMI and high arousal index was supported by our findings regarding the statistical significance of PLMI and arousal index (*p* = 0.002, *p* < 0.001, respectively, Table 3). The extent to which this phenotype affects long-term cardiovascular and cerebrovascular health, as supported by our findings, requires more investigation with covariate analysis of established OSA risk factors such as BMI, diabetes mellitus, hypertension, and smoking and has yet to be completely defined.

Several well-established risk factors have been characterized as contributing to OSA. Of these factors, the most strongly associated are elevated body mass index, diabetes mellitus, hypertension, male gender, age, and smoking [24]. Obesity is considered one of the strongest predictors of OSA due to mechanical obstruction risk increasing with increased excess tissue surrounding the upper airway [5]. A high arousal index, which reflects the frequency of sleep disruptions per hour of sleep, has been associated with increased BMI as a result of greater upper airway collapsibility, which leads to more frequent respiratory events and consequent sleep arousals [24]. A higher arousal index was observed in our findings to be increased in the CAD group. These risk factors all must be considered in observing differences in polysomnography parameters when establishing OSA phenotypes such as the high PLMI/high arousal index our findings sought to characterize. Further study into coronary artery disease risk associated with this OSA phenotype requires the addition of OSA risk factors to further define this phenotype and its associated risk profile.

There were a few limitations to this study. The first limitation is that many common OSA risk factors, such as hypertension, BMI, smoking, and diabetes, could not be controlled due to dataset collection limitations, which impact the analysis and interpretation of the results. These OSA risk factors are strong predictors of OSA and have been widely established in the literature. This limitation was addressed by propensity matching cases to comparative group patients by gender and age; however, further study is necessary to determine whether these risk factors play a role in the observed PLMI and Arousal Index differences between the CAD and Comparative groups. Another potential limitation is the smaller sample size of the study, which carries a risk of overfitting when performing multivariable analysis. This limitation was mitigated by the utilization of multiple statistical tests appropriate for sample sizes less than 50, such as the Shapiro-Wilk test. The lack of demographic diversity in patients is an additional limitation. The sample consisted of Egyptian patients, which may not account for demographic variation among different groups in polysomnographic trends. While this may be a limitation to the generalizability of the data, both the comparative and control group being of the same ethnic background strengthens the comparison between groups, as both groups are more likely to have similar environmental factors. An additional limitation is the nature of the comparative group and CAD group; the CAD group data was gathered prospectively, while the comparative group was a historical retrospective cohort. This design is a limitation that affects the comparability of the data and may potentially add bias to the study. This retrospective cohort was utilized to allow for a larger comparative sample to perform a 3:1 propensity match. One other limitation is that body position may influence AHI, which could subsequently impact the results and conclusions drawn in this study. American Academy of Sleep Medicine (AASM) Standard Manual for scoring sleep-associated events was utilized in the data collection of this study to minimize variability in recording procedures across patients. Lastly, the final limitation is that the comparative group is a group of non-CABG patients. Additional study into OSA comparing two groups of patients referred for CABG could further elucidate the potential connection between OSA and cardiovascular disease risk. Regardless of these limitations, the differences between abnormal sleep parameters in the CAD group and the comparative group in the PLMI, arousal index, and AHI parameters suggest a potential connection to a specific high PLMI and arousal index OSA phenotype that is most at risk for cardiovascular disease. If a CAD OSA group could, in the future, be compared to an OSA comparative group without CAD, this may provide defining evidence of a specific OSA phenotype predisposed to CAD and other cardiovascular disease risks.

## Conclusion

It is estimated that around one billion people have OSA, but the full extent of its systemic effects on the body remains unclear. In recent years, there has been an emergence of analysis on specific OSA phenotypes that are defined by patient symptoms, response to therapy, health outcomes, and quality of life. This analysis aimed to build upon the characterization of a high PLMI/high Arousal Index phenotype associated with increased cardiovascular disease risk by examining polysomnographic parameters in CAD patients undergoing preprocedural screening before CABG. Our findings, combined with existing literature, show that a high PLMI/high Arousal Index phenotype of OSA may be a potential factor in CAD and cardiovascular disease risk associated with OSA. Further analysis that considers OSA risk factors such as body mass index, diabetes mellitus, smoking history, and hypertension is necessary to define this phenotype and further evaluate any risks related to it.

## Supplementary Information

Below is the link to the electronic supplementary material.

Supplementary Material 1 (DOCX 16.5 KB)

## Data Availability

The data are available upon reasonable request from the corresponding author.
