# Supplementary material for: Polysomnographic arousal index and periodic limb movement index in patients undergoing coronary artery bypass grafting: an exploratory case-comparison study
Source: Sleep Breath. 2026 Aug 6;30(4):233. doi: 10.1007/s11325-026-03779-9 (PMC13447470; doi:10.1007/s11325-026-03779-9)
Supplement: Supplementary file 1 [file 11325_2026_3779_MOESM1_ESM.docx]

Supplemental Table 1. Subject Characteristics and Polysomnography Parameters in CAD Group and Comparative Group

| Subject Characteristics | CAD group (n=30) | Comparative group (n=197) | p |
| --- | --- | --- | --- |
| Age | 56 (±**7.17**) | 49 (±**7.22**) | 0.035* |
| Gender (male) | 24 (80%) | 91 (46%) |  |
| PSG Parameters | | | |
| Sleep Efficiency % | 93 (82-99) | 90 (47-100) | 0.086 |
| Sleep Onset, min | 5.93 (1.00-16.50) | 9.9 (0- 44.0) | 0.053 |
| AHI, N/hr | 18.3 (0.0-70.3) | 9.2 (0-63.0) | 0.014 * |
| Arousal Index, N/hr | 19.1 (2.5-46.7) | 9.5 (0.0-30.0) | <0.0001* |
| PLMI, N/hr | 14.0 (0.5-31.6) | 4.0 (0.0-40.8) | <0.0001* |

Abbreviations: PSG, polysomnography; PLMI, peripheral limb movement index; CAD, coronary artery disease; AHI, apnea-hypopnea index

Supplemental Table 2. Multicollinearity statistics

|  | Arousal Index | AHI | PLMI |
| --- | --- | --- | --- |
| Variance Inflation Factor | 1.252 | 1.244 | 1.034 |
| Tolerance | 0.799 | 0.804 | 0.967 |

PLMI, periodic limb movement index; AHI, apnea-hypopnea index
